# Experimental and Computational Studies on the Basic Transmission Properties of Electromagnetic Waves in Softmaterial Waveguides

**DOI:** 10.1038/s41598-018-32345-x

**Published:** 2018-09-14

**Authors:** Jingjing Xu, Yuanyuan Xu, Weiqiang Sun, Mingzhi Li, Shengyong Xu

**Affiliations:** 1 Department of Electronics, School of Electronics Engineering and Computer Science, Peking University, Beijing, 100871 P. R. China; 2 Key Laboratory for Physics and Chemistry of Nanodevices, Peking University, Beijing, 100871 P. R. China

## Abstract

Conventional waveguides are usually made of metallic materials, and they are effective pathways for the transmission of electromagnetic waves. A “*Softmaterial waveguide*”, by contrast, is supposed to be made of dielectric material and ionic fluids. In this work, by means of both experiment and computational simulation we examined one kind of softmaterial waveguide, which has the configuration of ionic fluids filled in and out of a dielectric tube. We investigated configurations with varied parameters, i.e., tube thickness from 0.2 mm to 5.0 mm, tube length of 2.0–12.0 cm, ionic concentration covering 4 orders of magnitude from 0.0002–2.0 mol/L, frequency of 10 Hz to 100 MHz for sine wave excitations, pulse duration of 5 ns to 100 ms for excitation pulses. We also mimicked the myelin sheath structure in myelinated axons in simulation. Both experimental and simulation results consistently showed a clear confinement effect for the energy flux of transmitting electromagnetic waves inside the dielectric tube, strongly supporting the model of *softmaterail waveguide*. The results revealed that the *softmaterial waveguide* had a low-pass nature, where the intensity of transmitted signals saturated at a duration of 10–100 μs for pulses, or cut off at frequency of 10–100 kHz for sine waves. And, the transmission efficiency increased with the thickness of the dielectric layer, as well as ion concentration of the solution. The results may help for a better understanding various electrical communication behaviors observed in biosystems, where a natural lipid membrane with bilateral fluids was suggested as the efficient pathway for pulsed neural impulses in a way similar to soliton-like electromagnetic pulses transmitting in a softmaterial waveguide.

## Introduction

Studies on the electrical phenomena in nerves could be traced back to 1890s^1^. Both techniques and theories in this field have been well developed since 1950s after the inventions of voltage clamp^2^ and patch clamp^3^. Currently the common theory for the neural communication was proposed by Hodgkin and Huxley in 1952, in which the nerve impulses such as action potentials propagate by electric current running along axons, in a way similar to that of an electric current running in electric cables^4^. In this model, the axon membrane (or myelin sheath in a myelinated axon) only serves as an electric insulating layer separating ionic fluids in and out of the membrane, and the dielectric property of this layer determines the transmembrane capacitance. However, this theory faces difficulties in describing so-called “*saltatory conduction*” in myelinated axons and some latest experimental phenomena. For example, the collisions of two impulses propagating in orthodromic and antidromic directions in the giant axons of earthworms and lobsters did not result in the annihilation of the two signals, contrary to the notion on the refractory period in the Hodgkin – Huxley model^5^. This contradiction has been repeatedly found later by other researchers^6^. Heimberg and Jackson *et al*. stated that nerve signals were electromechanical solitons in the form of dilatational waves, and transmitted via cell membranes^7,8^. The electromechanical solitons were assumed to be generated by the transmembrane ionic currents, in which the Joule heat by the electric current resulted in a phase transition in the local membrane. This model has supporting evidences from previous experiments and computational simulations that there were changes of local heat and membrane displacement along with the action potential process^9–13^. The model could explain the non-annihilation condition of the two signals in collision^5,6^, by describing the nerve signals as waves for the first time. Nevertheless, it encounters problems in explaining the “*saltatory conduction*” in myelinated axons and the neural transmission in poikilotherm^14^.

The soliton-like electromagnetic (EM) pulse model was proposed in 2012^15^. The model stated that the electric nerve impulses were actually pulsed electromagnetic waves, which were generated by transmembrane transient ionic currents according to Maxwell’s equations, and the pulsed EM waves propagated along “*softmaterial waveguide*” pathways, which were constructed with cell membranes and cytoplasm fluids at both sides of the membranes. The observed phenomenon of no-delay in transmission of electric signals at electrical synapses was explained with the concept of coupling between two softmaterial waveguides. Signal transmission from synapses to muscle fibers and “*saltatory conduction*” between two nodes of Ranvier in myelinated axons could also be described well with this model. Recently, this model was extended to explain EM communication phenomena in unicellular organisms, plants and electric eels^14^, where three roles of lipid membranes in electric power and EM communication of biosystems were emphasized^16^. Andrea Zangari *et al*. suggested the possibility of EM radiation in axons by the computational modelling in 2018^17^. They found that lights with the wavelength below 1600 nm were likely to propagate in myelinated axons with the assumption that the ionic currents at the Ranvier node behave like an array of nano-antennas. The results positively support the soliton-like EM pulse model.

A “*softmaterial waveguide*” is a waveguide made of soft maters. It is one of the most basic elements in soliton-like electromagnetic EM pulse model, however, its detailed properties are not clear to date^15^. Soft matter comprises a variety of physical systems which are deformed or structurally altered by thermal or mechanical stress, including colloids, polymers, granular materials, liquid crystals, and biological materials^18–20^. Dr. Gennes was honored with a Nobel Prize in physics due to his study on soft matters^21^. In a general definition, a waveguide is a device that may help to minimize the loss of EM energy by restricting expansion of the EM wave in one or two dimensions^22–24^. Conventional EM waveguides for microwaves and radio frequency signals are usually made with metallic materials. When dimensions for the cross-section of a hollow metal pipe match a portion of the wavelength of an EM wave, it may lead to transmission modes of Transverse Electric Field (TE) or Transverse Magnetic Field (TM)^25,26^. Dielectric waveguides employing solid dielectrics, such as optical fibers, are also typical waveguides for lights in long-distance information transmission^27–29^. By contrast, *softmaterial waveguides* are supposed to form when a dielectric plate or tube is merged in an ionic fluid, but with a lower transmission efficiency than that of metal waveguides^15^. In biosystems such as an axon, its membrane (phospholipid bilayer) is a good dielectric layer of 2–3 nm in thickness. Together with ionic fluids at its both sides, the whole structure forms a softmaterial waveguide^14–16^. The myelin sheath of a myelinated axon, with a thickness up to a few microns, is made of many layers of lipid bilayers, which is a uniform and better dielectric layer in the view of softmaterial waveguide. By using a radio signal emitter and an antenna receiver, Li *et al*. obtained preliminary results showing that a structure of liquid - plastic tube - liquid qualitatively behaved like a waveguide^30^. In a recent work, it was found that visible lights could pass through aggregated channels of cyanobacteria with deeper penetration length in seawater than in control samples without the cyanobacteria, where a chain of the cyanobacteria might serve as a waveguide^31^.

In this work, by means of experiments and computational simulations at the millimeter and centimeter scales, we investigated properties of the softmaterial waveguides constructed with dielectric tubes and ionic fluids, one kind of many possible softmaterial waveguide configurations. We studied the influences of several factors on the properties, such as wave frequency or pulse duration for the excitation signals, length and thickness of the dielectric layer, and the concentration of the ionic fluids. The results from simulations and experiments were consistent with each other, confirming that softmaterial waveguides worked well in confining electromagnetic waves within the dielectric layer.

## Results

### Experimental results

#### Confinement effect of softmaterial waveguides

In this part, the transmission efficiency refers to “V_pp_/V_ref_”, where V_pp_ is the amplitude of the signals passed through the device samples under test, and V_ref_ is the amplitude of the reference signal. The attenuation length L_0_ is defined as the propagation distance where the amplitude of the signal drops to 1/*e* of the original value. L_0_ is a measure for the confinement effect of a waveguide: Compared to a bad waveguide, a good one confines transmitting EM energy more efficiently within its dielectric layer so that an EM wave can propagate longer distance within the dielectric layer at the same attenuation. In the case of this work, the dielectric layer of a device under test is the polyethylene (PE) or polystyrene (PS) tube.

**Figure 1:**
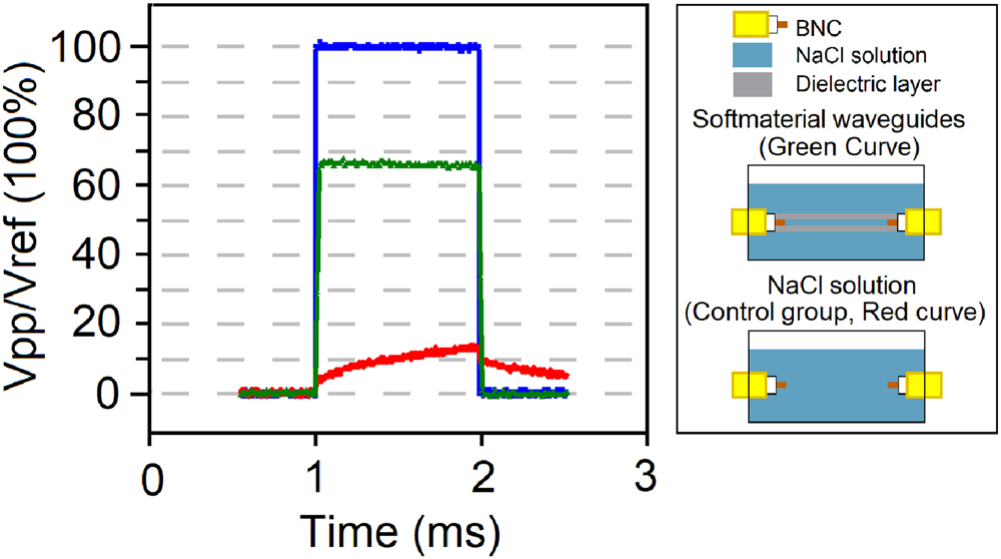
The confinement effect of a softmaterial waveguide for EM pulses. The left panel presents the measured amplitudes of pulsed EM signals after they transmitted through a coaxial cable as the reference (blue), a softmaterial waveguide structure (green), and a control sample with pure 0.1 mol/L NaCl solution. The softmaterial waveguide sample was made of an 8.0 cm long, 1.0 mm thick PE tube with 0.1 mol/L NaCl solution filled in and out of the tube. The right panel illustrates device configurations for the measurement.

In our experiments, we investigated the transmission efficiency and L_0_ for tube-based samples with a variety of different parameters, i.e., tube length from 2.0 cm to 12.0 cm, tube thickness from 0.2 mm to 5.0 mm, ionic concentration from 0.0002 mol/L to 2.0 mol/L, etc. For excitation signals, we investigated the results for sine wave exciation with frequency from 10 Hz to 100 MHz, and for EM pulse excitation with pulse duration from 5 ns to 100 ms and varied pulse repeating frequency. Figure 1 presents general result, showing the basic confinement nature for EM waves of a plastic tube-based sample merged in a NaCl solution. Here the excitation signals were square EM pulses with the duration of 1.0 ms at a repeating frequency of 100 Hz. The absolute peak-peak voltage was 2.0 V, and both the lead/trail edges were set to 2.5 ns. The EM pulses transmitted through an 8.0 cm long, 1.0 mm thick PE tube in a 0.1 mol/L NaCl solution that filled both the inner and outer space of the tube, and resulted in the green curve at the other end of the tube. This softmaterial configuration showed an EM transmission efficiency of ~68% as compared to the reference signal (shown in blue). And, the transmitted signal remained a good square pulse shape. By contract, the excitation EM pulses passing through pure NaCl solution in the control sample resulted in a distorted signal shape and much lower transmission efficiency, as shown in the red curve.

#### “Low-pass” property of softmaterial waveguides

Besides the basic confinement effect, which is the fundamental nature of an EM waveguide, we also observed several special characteristics of the tube-based “*softmaterial waveguide*” samples. The transmission efficiency for these samples was strongly dependent on excitation frequency or pulse duration, tube thickness, and ion concentration, etc.

**Figure 2:**
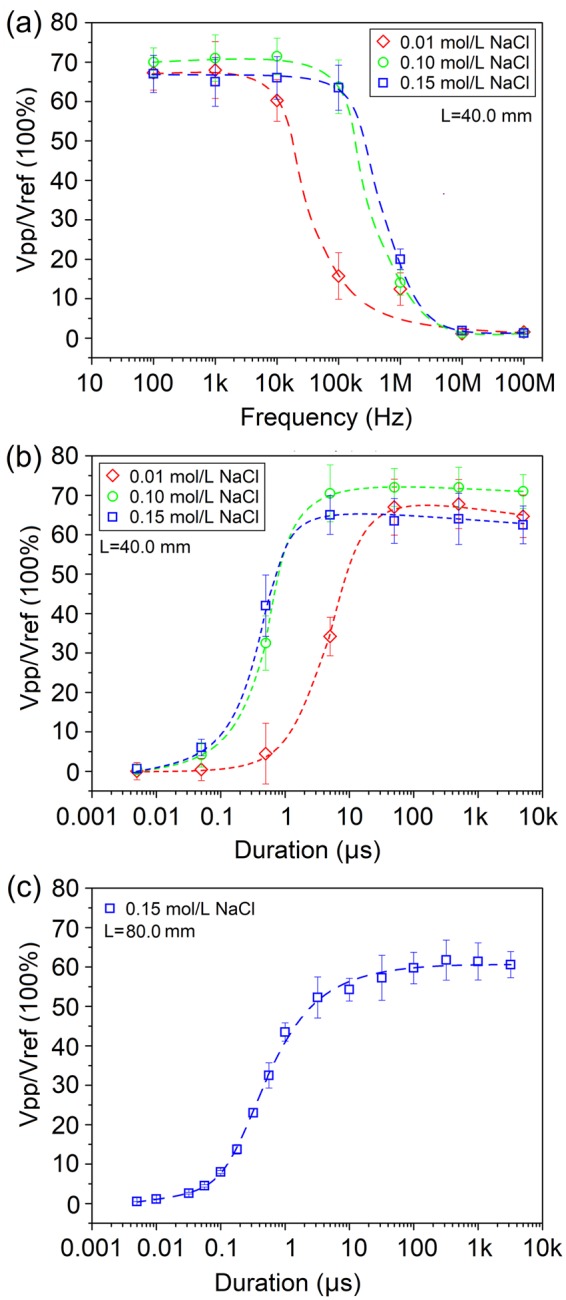
Three sets of measurement data showing the dependences of transmission efficiency on (**a**), the frequency of EM sine waves, and on (**b**) and (**c**), the pulse-duration of square EM pulses. For (**a**) and (**b**), identical PS tubes of 4.0 cm long and identical sample container were used for varied concentration of NaCl solution, and each device was tested multiple times in two excitation modes. For (**c**), an 8.0 cm long tube was used.

Figure 2 presents three sets of typical measurement results for the frequency and pulse-duration dependence of the tube-based softmaterial waveguide samples. Figure 2(a,b) show cross-over testing results for PS tube-based samples in 0.01, 0.10 and 0.15 mol/L NaCl solutions, where for each measurement both in and out of the tube the ionic solution had the same concentration. The PS tubes used in these samples had the same dimensions of 4.0 cm in length, 6.0 mm for outer diameter, and 2.0 mm for the thickness of the tube wall. In Fig. 2(a), the data were taken from the samples with sine excitations at 7 different frequencies, namely 100 Hz, 1.0 kHz, 10.0 kHz, 100.0 kHz, 1.0 MHz, 10.0 MHz and 100.0 MHz, respectively. The measured transmission efficiency started to drop sharply at frequency of 10 kHz–100 kHz, and reduced to almost zero at frequency of 10 MHz to 100 MHz. For pulsed EM excitations, as shown in Fig. 2(b), the data were taken from the samples with pulsed excitations at 7 different pulse durations, namely 5 ns, 50 ns, 500 ns, 5 μs, 50 μs, 500 μs and 5 ms, respectively. The measured transmission efficiency increased sharply at pulse duration of 100 ns to 1 μs.

In Fig. 2(c), we plotted more detailed measurement results of a longer PE tube (8.0 cm long, 1.0 mm thick) in 0.15 mol/L NaCl solution for the pulse duration dependence. From 5 ns to 3.2 ms, 16 varied frequencies were applied. The cure is smooth and saturates for pulse duration at the range of 10–100 μs and longer. Again, it confirms the low-pass nature of the tube-based softmaterial waveguides.

According to law of Fourier transformation, pulses with wider duration contain more low-frequency signals. Therefore, the results shown in Fig. 2(a,b) are consistent with each other, both revealing the same low-pass nature of the devices under test. In particular, for this set of samples, they have no confinement effect on the EM pulses with duration less than 0.1 μs or EM sine waves with frequency higher than 10 MHz. Within the measurement errors, the saturated transmission efficiency varied in the range of 65–75% with different solution concentration. And a sample with lower concentration (here, 0.01 mol/L) resulted in a lower cut-off frequency, or longer saturation pulse duration.

**Figure 3:**
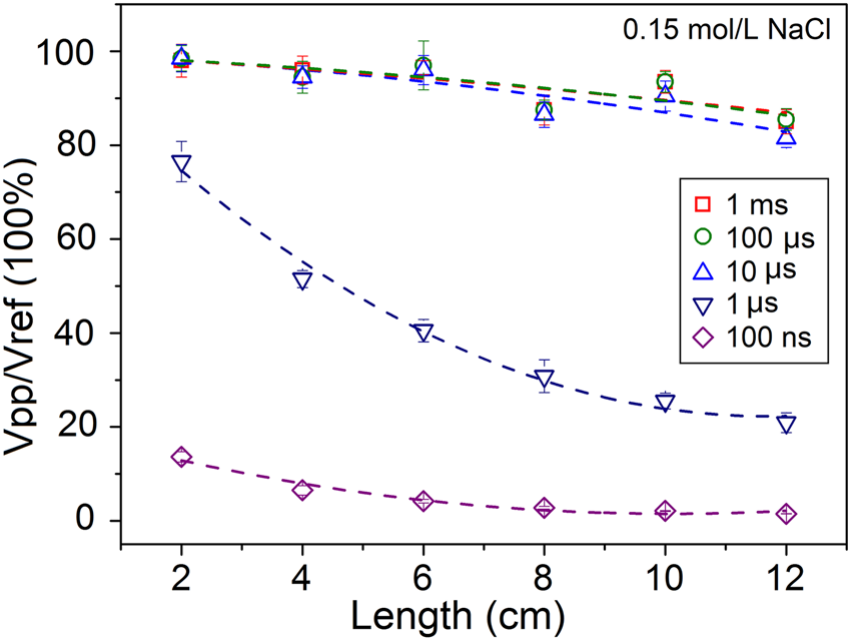
Measurement results of transmission efficiency on the tube length and excitation pulse duration from an 8.0 cm long softmaterial waveguide in 0.15 mol/L NaCl solution. Dashed fitting guidelines show the trends of the data.

Next, we examined the dependence of transmission efficiency on the length of the softmaterial waveguides to confirm the low-pass characteristics. Figure 3 presents a set of measurement data taken from samples with identical PS tubes with outer diameter of 6.0 mm, tube thickness of 2.0 mm but tube length from 2.0 cm to 12.0 cm. The inner and outer solution was set the same as the saline solution. EM pulses with 5 different pulse durations, namely 100 ns, 1 μs, 10 μs, 100 μs and 1 ms were used as excitation signals. One sees two features in these results. One is that transmission efficiency decreases with the tube length, which is naturally expected. The other is that transmission efficiency also decreases with the pulse duration. This is consistent with the results shown in Fig. 2(b,c). The attenuation lengths L_0_ for the 5 different pulse durations are calculated as 251, 229, 131, 10 and 6.1 cm, respectively.

#### Dependence of transmission efficiency on tube thickness and ion concentration

**Figure 4:**
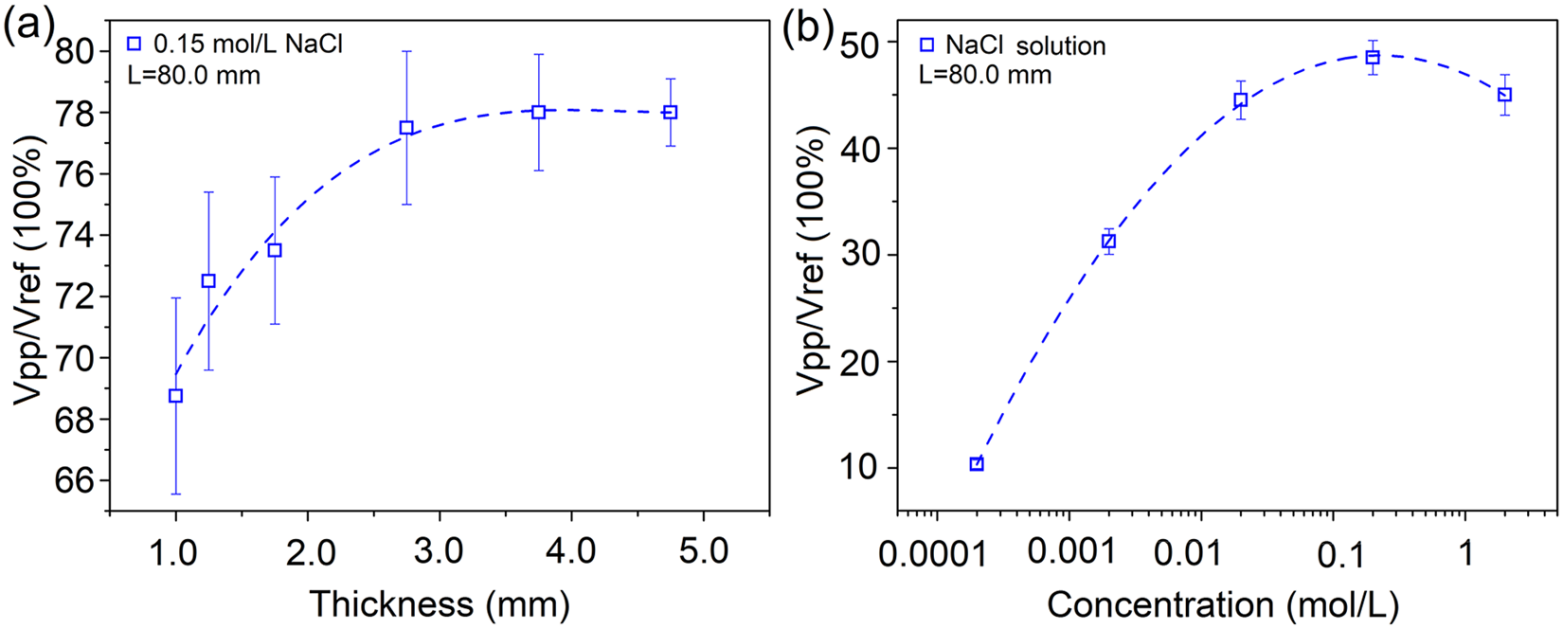
Dependence of transmission efficiency on (**a**), the tube thickness of sample, and (**b**), ion concentration of NaCl solution used for the sample under 320 μs long EM pulses. For (**a**), all tubes had the same inner diameter of 2.2 mm. For (**b**), the tube had a thickness of 0.2 mm and diameter of 6.0 mm.

Figure 4 plots two sets of measurement data taken from samples with tube thickness and ion concentration as the variables, respectively. EM pulses with duration of 0.32 ms were used as excitation signals. Figure 4(a) shows data for which the saline was used as the inner and outer solutions, and the tubes all had the same length of 8.0 cm and the same inner diameter of 2.2 mm. The raw data had large errors, indicating that the tube thickness was a sensitive factor for the testing results. Within the measurement error, we observed a rough trend that the transmission efficiency increased with the tube thickness in this bunch of samples. Figure 4(b) shows some typical results taken from some samples with tube thickness of 0.2 mm and tube diameter of 6.0 mm. Here, the transmission efficency roughly increases with the ion concentration of the NaCl solution.

#### Computational results

**Figure 5:**
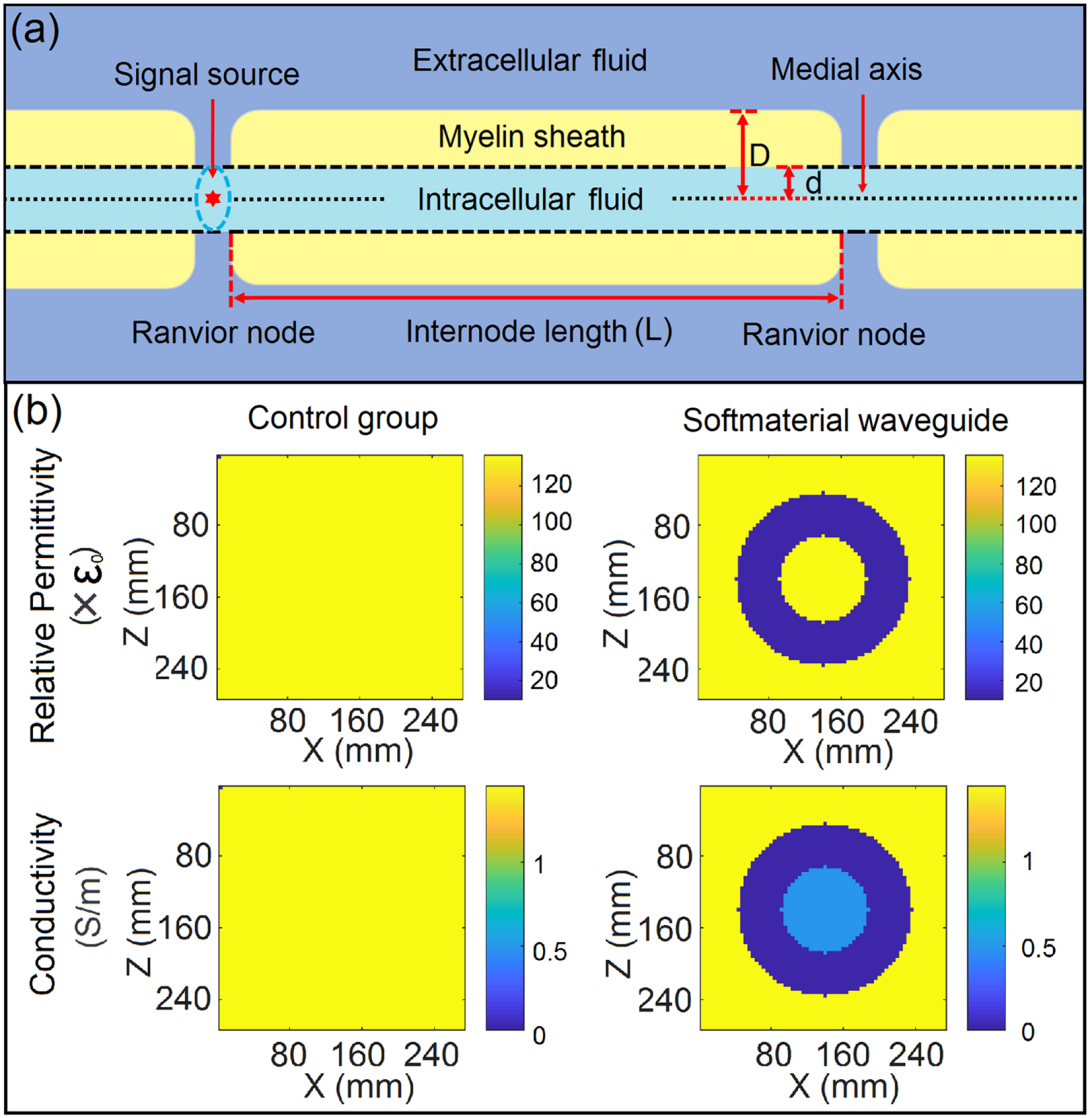
(**a**) Schematic illustration of the lengthwise section of the model simplified from a myelinated axon, in which the internal diameter d, external diameter D and internode length L were set at 48.0 mm, 96.0 mm, 240.0 mm, respectively. (**b**) The sectional views of the distributions for relative permittivity and conductivity in control group and softmaterial waveguides.

After the verification of computational methods, simulating the transmission of EM waves in the softmaterial waveguide were conducted. In biosystems, neural pulses transmit much faster in myelinated axons than in unmyelinated axons by a factor of 10–100^32,33^. In myelinated axons, the thickness of the myelin sheath is about a few microns, which are roughly 1000 times thicker than the membrane for an unmyelinated axon^32,33^. It is proposed that the myelin sheath, a long, thick and hollow dielectric tube, plays the key role and serves as the main transmission path for pulsed EM waves that are generated at the transmembrane protein channels^14,15^. The myelin sheath and the fluids in and out of the sheath form a softmaterial waveguide^15^. To verify this model with computational simulation, we simplified the structure of a segment of a myelinated axon into a model shown in Fig. 5(a), where the intracellular fluid and extracellular fluid serve as the solutions and the myelin sheath serves as the dielectric layer. The internal diameter d, external diameter D and internode length L were set at 48.0 mm, 96.0 mm and 240.0 mm, respectively.

The parameter setting for the softmaterial waveguide structure and the control group (in which the setup was made of homogeneous solution only, without softmaterial waveguide structure) are illustrated in Fig. 5(b), which gives the sectional views of the distributions of relative permittivity and conductivity in two setups. The relative permittivity of the dielectric layer, inner solution and outer solution are 10.3, 136.0, and 136.0 respectively, while their respective electrical conductivities are 0, 0.487, and 1.454 S/m according to the data from references^34,35^.

The time-domain pulsed signals are the superposition of a variety of single-frequency waveforms due to the Fourier transform. As a result, we utilized single-frequency sine waves as excitation sources in the analysis of steady-state distribution and energy loss of electromagnetic fields in softmaterial waveguide model, and employed time-domain Gaussian pulses for analyzing their transient transmission properties in this waveguide model.

#### Sine waves as source signals

**Figure 6:**
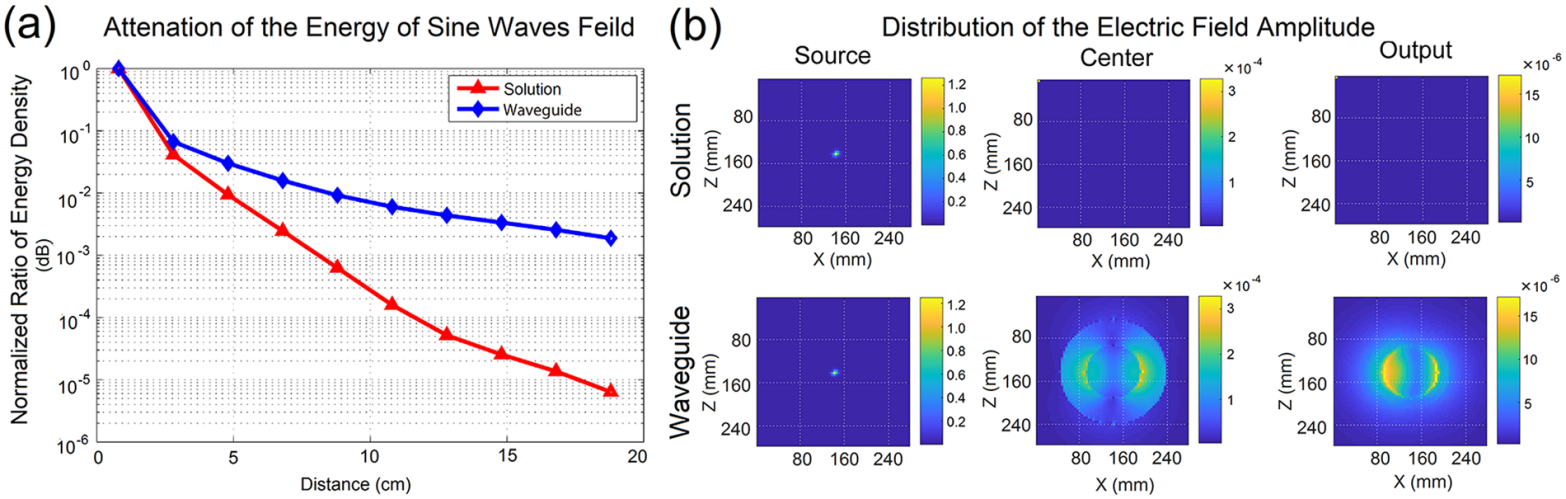
The verification of confinement effect for softmaterial waveguides. (**a**) Comparison of the attenuated curves of normalized energy density for EM signals transmitting in the softmaterial waveguides and the solution along the axial direction. (**b**) The electric field amplitude distribution on cross section at the source, center and output position respectively along the axial in the two setups.

Sine waves with the width of λ = 1.44 m were mounted at the medial axis of the modeled axons as excitation sources. The transmission properties along with the distance in softmaterial waveguides and pure solutions (control group) were calculated with FDTD method, and shown in Fig. 6.

Figure 6(a) illustrates that the normalized energy density of EM signals in the softmaterial waveguide decreases slower when compared to that in the solution only, which demonstrates the confinement effect of softmaterial waveguides on EM waves. The electric field amplitude distribution on cross sections at three different positions is shown in Fig. 6(b), which indicates that there is the same electric amplitude distribution at the source position for the two setups. In comparison, at the central position, there is hardly any electric field in the pure solution, while in the softmaterial waveguide there is a quite strong electric field within the dielectric layer and the inner solution. A stronger electric field is found at their interface. At the output position, the electric field at the interface seems to be stronger but to occupy a smaller area in the waveguide, while there seems no electric field as well in the solution. The results from Fig. 6(b) are consistent with the results in Fig. 6(a), demonstrating that the softmaterial waveguide can improve the transmission efficiency of EM waves when compared to the pure solution, where the EM waves disperse rapidly.

#### Gaussian pulse as source signals

**Figure 7:**
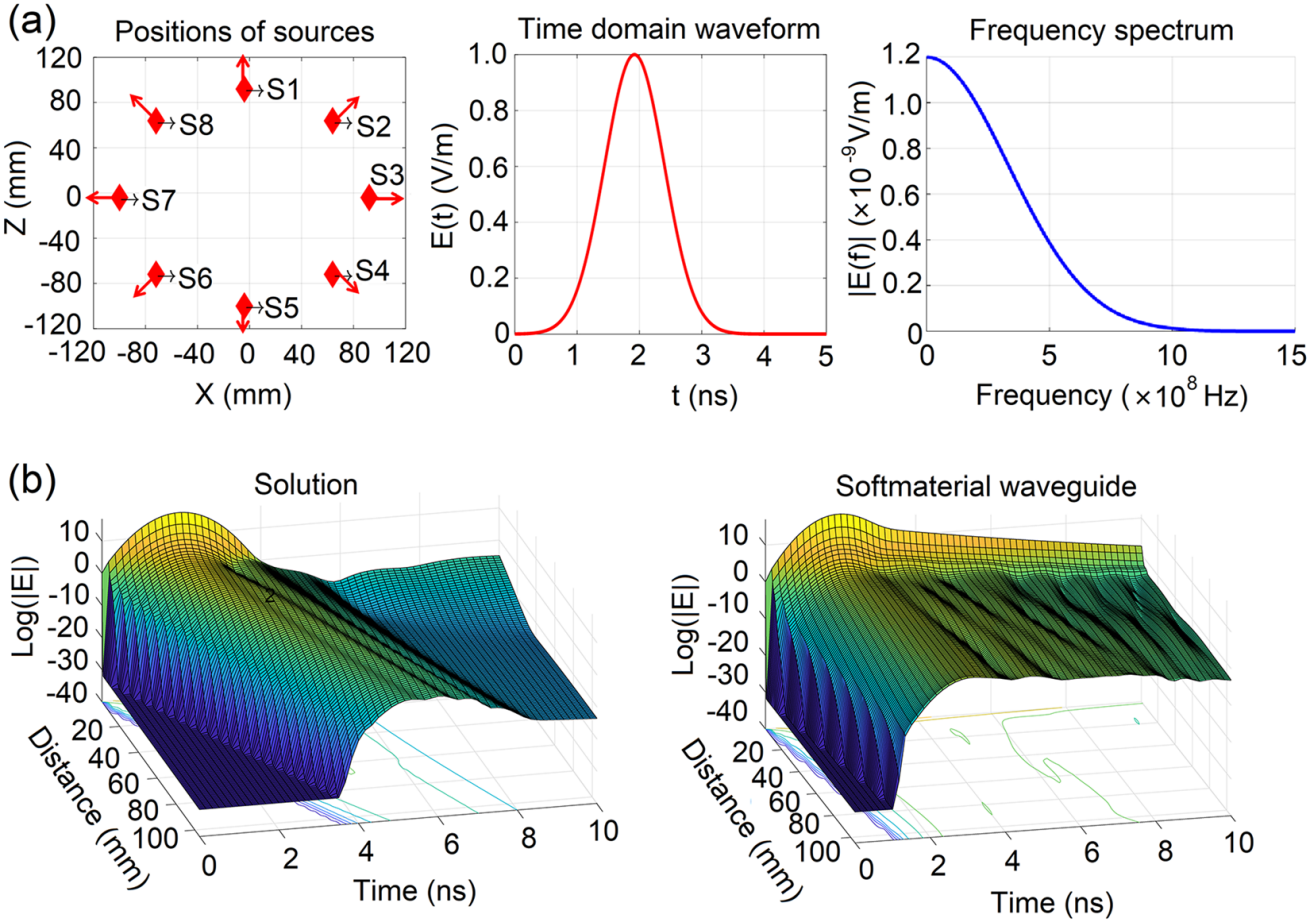
The setups and results from the simulation on Gaussian signals. (**a**) The position, time domain waveform and frequency spectrum of Gaussian signal sources; (**b**) The propagation properties of Gaussian pulses in solution (left, the control group without waveguides) and in softmaterial waveguides (right).

We replaced sine waves with Gaussian pulses to simulate the transmission properties in softmaterial waveguides. The Gaussian pulse is defined by the following expression:

1 $$E(t)=\exp (-\frac{4\pi {(t-{t}_{0})}^{2}}{{\tau }^{2}}),$$

where τ = 2.40 × 10^−9^ s and t_0_ = 1.92 × 10^−9^ s when taking the computing resource into consideration. Eight sources of the same Gaussian pulse polarizing along the radial direction were placed at the dielectric layer uniformly at the same cross section, shown in Fig. 7(a). The spatiotemporal propagation of the Gaussian pulses in the softmaterial waveguide and solutions was simulated. Here, the form, the polarizing direction and the source position of the signals resembled those of the signals in nerves.

The results in Fig. 7(b) show that, initially only partial space was motivated by the signal sources. Then the signals propagated ahead along the axial direction until the whole space was motivated. Compared to in the control group without waveguides, the motivation period in the waveguide was much shorter, less than 2 ns, which meant that the existence of the waveguide sped up the transmission of signals. Furthermore, the signals in waveguide were found to attenuate much less for the same transmission distance, which demonstrated the confinement of the softmaterial waveguides on Gaussian pulses.

Here we simulated the transmission properties of EM waves including sine waves and Gaussian pulses in softmaterial waveguides modeled on the myelin sheath. Compared to the pure solution, softmaterial waveguides can confine EM waves, thus reducing the energy loss of waves and improving their transmission efficiency.

## Discussion

**Figure 8:**
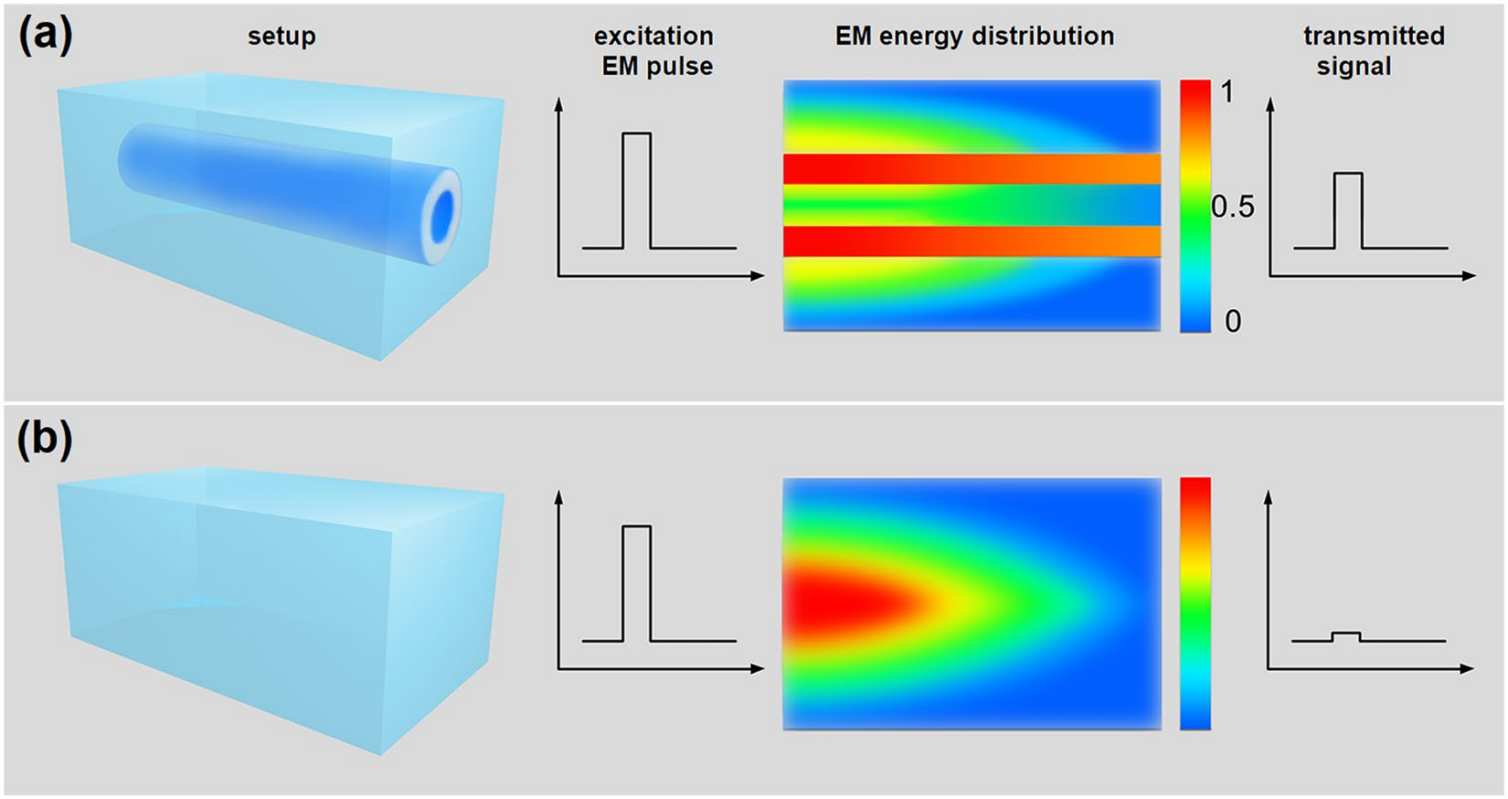
A schematic diagram of the confinement effect on EM waves with tube-based softmaterial waveguide. (**a**) The device is made with a dielectric hollow cylinder merged in an ionic solution. When EM pulses (or sine waves) penetrate the devices, the dielectric layer well confines the EM energy, resulting a high transmission efficiency. (**b**) A control sample with pure ionic solution of the same concentration. EM pulses transmitted through this device result in a much lower transmission efficiency.

Our experimental and computational results consistently demonstrated that a cylindrical shape plastic (dielectrics) tube, filled with ionic solutions in and out of the tube, formed a device that fulfilled the concept of a “*softmaterial waveguide*”. As conclusively illustrated in Fig. 8(a), such a device could efficiently confine the EM wave energy within its dielectric layer along the longitudinal propagating direction. With properly chosen parameters, such as the tube thickness and ion concentration, a transmission efficiency of 60–80% was achievable over a distance up to 12.0 cm. By contrast, if the EM waves transmitted through a control sample, which had the same sample configurations but without the dielectric tube, the transmission efficiency was much lower, as shown in Fig. 8(b).

When EM waves propagate in traditional metallic EM waveguides, their electric field almost totally reflects at the metallic surface. In softmaterial waveguides, however, the propagating EM energy leaks partially at the interface between the dielectric layer and fluids. Therefore the transmission efficiency of softmaterial waveguides is always lower than that of metallic waveguides.

The low-pass nature revealed in our experiments has an important impact for understanding the transmission phenomena of neural impulses in biosystems. In both our experiments and simulations, pulsed EM signals were applied to mimic the EM signals in nerve systems. These pulsed EM signals are not EM solitons, yet they might share some similarity with EM solitons in their transmission behavior in softmaterial waveguides. We found in this work that when the pulse duration was longer than 10–100 μs, the amplitude of transmitted EM pulses saturated at almost a constant value, as shown in Fig. 2(b,c). In biosystems, the durations of neural impulses are mostly around 1 ms, and sometimes a bit longer. The collective electric signals of a brain (resulting from millions and billions of neurons, dendrites and axons) usually have basic frequency of 5–50 Hz, corresponding to the period of 20–200 ms. And recently, a noninvasive deep brain stimulation method was developed via a temporally interfering electric field targeting on the neurons in the hippocampus of living mice. It demonstrated that two sources of high frequency electric fields could not stimulate the neurons on the way to the target, but the low frequency electric field resulting from the interaction of the two coherent high frequency electric fields could trigger local neurons deep in the brain^36^. These facts might have profound interconnections. If the nature did developed a softmaterial waveguide system (e.g., fluid-membrane-fluid in axons and normal cells) that are in favor of long-duration EM pulses, and transient currents did generate soliton-like EM pulses as previously proposed^14–16^, then the sensors or ion channels in the neurons and axons should also favor these long-duration, low-frequency EM pulses. Our results in this work indeed strongly support such a scenario, though it still need direct measurement performed in live biosystems to prove it.

The structures of softmaterial waveguide samples tested in this study are similar to those of axons, except for the difference in their dimensions. Our sample had larger thickness than that of unmyelinated axon (2–3 nm) and myelin sheath (1–5 μm) by a factor of several orders of magnitude. The measured and simulated sample length was also much longer than the average spacing of 10–100 micron between two ion channel clusters in myelinated axons and up to a few millimeters between neighboring nodes of Ranvier in myelinated axons^37^. This is due to the limitation in experiment setup and in simulation capacity (see Supplementary Information). However, there seems to be no dimensional limit for Maxwell equations. A theoretical discussion stated that even at the nanoscale, EM waveguides could work well, e.g., in graphene based structures^38^. A more recent simulation stated that there were three kinds of potential waveguides capable of serving as the transmission paths for EM waves in optic nerve axons, one of which was composed of the myelin sheath and the bilateral fluids^17^.

We showed that the transmission efficiency of pulsed EM signals increased with the thickness of dielectrics (Fig. 4(a)). This may help to understand the role of myelin sheath, which has many bilayers of lipid membrane and a thickness of a few microns. This thick dielectric sheath makes myelinated axon a better EM waveguide than the unmyelinated axon, and results in a longer attenuation length. In fact, “internode length” could be as long as 0.3–2.0 mm^39–41^, sometimes reaches a length of 7.0 mm^37^, longer than the average spacing between neighboring ion channel clusters in unmyelinated axons by a factor of 100. Our results are consistent with previous evidences that the conduction velocity was positively related to the thickness of the myelin sheath^42^. However, some other literatures demonstrated that the conduction velocity in myelinated axons reached the maximum when the ratio of internal diameter to external diameter tend to 0.6^43,44^, while some scientists reported it was sensitive to the internode length, i.e., the distance between nodes of Ranvier in myelinated axon^42,45^. The validity of softmaterial waveguide discussed in this work and the proposed soliton-like EM pulse model^14–16^ may also help to the “saltatory conduction” in myelinated axons^41,46–48^. Indeed, it is hard to set up a longitudinal voltage between two nodes of Ranvier for generating an ionic current along the axon, but it is easier to allow pulsed EM signals to transmit within the myelin sheath, a dielectric layer as revealed in this work.

In the softmaterial waveguides discussed here, both the dielectric layer and the bilateral solutions are homogeneous medium, while the conditions in biosystem are more complicated. Most dielectric layers, which are mainly lipid-bilayer based membranes, are inhomogeneous in organisms due to proteins, glycoproteins and water molecules embedded in them. The fluids in organisms including intracellular fluids and interstitial fluids are non-Newtonian fluids, consisting of a variety of subcellular organelles and biological macromolecules such as proteins, carbohydrate, steroids and so on. Therefore, the confinement for EM waves in a natural biosystem, *e.g*., axons, is expected to be weaker.

## Conclusion

In summary, by performing experiments and computational simulations we studied the transmission efficiency of continuous and pulsed EM waves when they propagated through a special kind of man-made *softmaterial waveguide* structure, i.e., a plastic tube merged in an ionic fluid. A variety of variables i.e., tube thickness from 0.2 mm to 5.0 mm, tube length of 2.0–12.0 cm, ionic concentration covering 0.0002–2.0 mol/L, frequency of 10 Hz to 100 MHz for sine wave excitations, and pulse duration of 5 ns to 100 ms for excitation pulses, were investigated. We also mimicked the myelin sheath structure of a myelinated axon in simulation. The results of both approaches were consistent with each other, showing that a dielectric tube with solutions filling its both sides worked well as an EM waveguide. It could confine the energy flux of an EM wave within its dielectric layer, resulting in a better transmission efficiency for the EM wave than that of a fluid without the tube. Such a tube-based *softmaterial waveguide* showed a low-pass nature that the intensity of transmitted signals saturated at a duration of 10–100 μs for EM pulses, or cut off at frequency of 10–100 kHz for sine waves. The transmission efficiency increased with the thickness of the dielectric layer and the ion concentration. The results may shed light for a better understanding various electrical communication behaviors observed in biosystems, where a natural lipid membrane with bilateral fluids was suggested as the efficient pathway for pulsed EM signals. Yet the size of samples under test in this work was much larger than that of the axons in biosystems. To verify model for soliton-like EM pulse propagating in natural softmaterial waveguides of a biosystem, it needs further investigations.

## Methods

### Experimental method

The testing devices for softmaterial waveguide samples were made from polyethylene containers. Two holes were drilled symmetrically on two opposite sidewalls of the polyethylene container with the distance of 2.0–12.0 cm. One bayonet nut connector (BNC) was fixed into each hole and sealed with waterproof glue. The connection part of each BNC was at outside of the plastic container. Polyethylene (PE) and polystyrene (PS) tubes, with a relative dielectric constant (ε_r_) of 1.0–1.5 for PS and 1.5–2.5 for PE, were used as the dielectric layer. Thickness of the tubes varied from 0.2 mm to 5.0 mm. For each sample, the core wires of two BNCs were inserted into the polystyrene tube, and waterproof glue was used to seal the contact region. Electrolyte solutions were filled both inside and outside of the tube. Devices in the control group had the same two BNCs and electrolyte solution in the container, but no plastic tube between them.

A dual-channel pulse function arbitrary generator (PFAG, Agilent 81150 A) was used to send source signals to one BNC of a device under test. A four-channel digital storage oscilloscope (DSO, Agilent DSO90404A) was applied to receive the signal from the other BNC of the device. Open-ended coaxial cables (RG174) were used for connection between the device and the instruments. The dual-channel generator puts out two of the same pulsed signals at the same time, with one serving as the reference signal for the oscilloscope, and the other as the original signal to sample devices.

Sine waves with different frequencies and pulsed square shaped signals with varied durations were employed here. For pulsed signals, the rising edge and falling edge outputted by the generator were both set at 2.50 ns.

### Computational method

Finite - difference time - domain method (FDTD) was utilized to simulate the activities of EM waves transmitting in softmaterial waveguides. The FDTD technique is a well-developed computational method to simulate problems electromagnetic (EM) fields with complex geometry and electromagnetic parameter distribution. In this work we set the spatial discrete step and time step to meet the following Courant Stability Condition:

2 $${v}_{p}{\rm{\Delta }}t\le \frac{1}{\sqrt{\frac{1}{{({\rm{\Delta }}x)}^{2}}+\frac{1}{{({\rm{\Delta }}y)}^{2}}+\frac{1}{{({\rm{\Delta }}z)}^{2}}}},$$

where ${\rm{\Delta }}x={\rm{\Delta }}y={\rm{\Delta }}z$, ${\rm{\Delta }}x=2{c}_{0}{\rm{\Delta }}t$, and *c*_0_ is the speed of EM waves in vacuum. In view of the limited computing resource we have, a uniaxial perfectly matched layer (UPML) was introduced as the absorbing boundary condition to restrict the computational domain. Our computational simulation program was first verified by comparing the simulated results of several special cases, such as the radiation of free space dipole, bistatic RCS (radar-cross section) of dielectric sphere, etc., with the curves of corresponding theoretical formulas. In Fig. S1 of Supplementary Information we presents a typical testing result, showing the simulated radiation of a free space dipole with our program and that of theoretical formula. The FDTD solution and analytical solution match well with each other, proving the validation of the program and accuracy of the FDTD method.

## Electronic supplementary material

Supplementary Information

## Data Availability

The datasets generated during and/or analyzed during the current study are available from the corresponding author on reasonable request.

## References

[1] Hodgkin AL (1937). Evidence for electrical transmission in nerve. Part I. Journal of Physiology-London.

[2] Frankenhaeuser B, Persson A (1957). Voltage Clamp Experiments On The Myelinated Nerve Fibre. Acta Physiologica Scandinavica.

[3] Fishman HM (1975). Patch voltage clamp of squid axon membrane. Journal of Membrane Biology.

[4] Hodgkin AL, Huxley AF (1952). A Quantitative Description Of Membrane Current And Its Application To Conduction And Excitation In Nerve. Journal of Physiology-London.

[5] Gonzalez-Perez A, Budvytyte R, Mosgaard LD, Nissen S, Heimburg T (2014). Penetration of Action Potentials During Collision in the Median and Lateral Giant Axons of Invertebrates. Physical Review X.

[6] Fillafer C, Paeger A, Schneider MF (2017). Collision of two action potentials in a single excitable cell. Biochim Biophys Acta.

[7] Heimburg T, Jackson AD (2007). On the action potential as a propagating density pulse and the role of anesthetics. Biophysical Reviews and Letters.

[8] Heimburg T, Jackson AD (2005). On soliton propagation in biomembranes and nerves. Proceedings of the National Academy of Sciences of the United States of America.

[9] Abbott BC, Hill AV, Howarth JV (1958). The positive and negative heat production associated with a nerve impulse. Proc R Soc Lond B Biol Sci.

[10] Abbott BC, Howarth JV, Ritchie JM (1965). The Initial Heat Production Associated With The Nerve Impulse In Crustacean And Mammalian Non-Myelinated Nerve Fibres. Journal of Physiology.

[11] Ritchie JM, Keynes RD (1985). The production and absorption of heat associated with electrical activity in nerve and electric organ. Quarterly Reviews of Biophysics.

[12] Tasaki I, Kusano K, Byrne PM (1989). Rapid mechanical and thermal changes in the garfish olfactory nerve associated with a propagated impulse. Biophys. J..

[13] Tasaki I, Byrne PM (1990). Volume expansion of nonmyelinated nerve fibers during impulse conduction. Biophys. J..

[14] Xu S, Xu J, Yang F (2016). The Roles of Membrane for Electrical Communication in a Biosystem. Neuroscience and Biomedical Engineering.

[15] Xue, J. W. & Xu, S. Y. Natural electromagnetic waveguide structures based on myelin sheath in the neural system, http://arxiv.org/abs/1210.2140 (2012).

[16] Xu J, Yang F, Han D, Xu S (2018). Phenomena of synchronized response in biosystems and the possible mechanism. Biochemical & Biophysical Research Communications.

[17] Zangari A, Micheli D, Galeazzi R, Tozzi A (2018). Node of Ranvier as an Array of Bio-Nanoantennas for Infrared Communication in Nerve Tissue. Scientific Reports.

[18] Daoud, M. & Williams, C. E. *Soft Matter Physics*. (Springer, 2007).

[19] Hamley IW (2000). Introduction to soft matter. Chemical Engineering Journal.

[20] Mashaghi S, Jadidi T, Koenderink G, Mashaghi A (2013). Lipid Nanotechnology. International Journal of Molecular Sciences.

[21] Gennes PGDSM (1992). (Nobel Lecture). Angewandte Chemie International Edition.

[22] Maxwell JC (1954). A Dynamical Theory of the Electromagnetic Field. Philosophical Transactions of the Royal Society of London.

[23] Oguchi, M. *et al*. *IEEE Standard Dictionary of Electrical and Electronics Terms*. Vol. 42 (IEEE, 1977).

[24] Harvey, A. F. *Microwave engineering*. (Addison-Wesley, 2006).

[25] Lioubtchenko, D., Tretyakov, S. & Dudorov, S. Millimeter-Wave Waveguides. **40**, 859–911 (2007).

[26] Chakravorty P (2014). Analysis of Rectangular Waveguides - An Intuitive Approach. Iete Journal of Education.

[27] Miller SE, Marcatili EAJ, Li T (1973). Research toward optical-fiber transmission systems. Proceedings of the IEEE.

[28] Kawachi M (1990). Silica waveguides on silicon and their application to integrated-optic components. Optical & Quantum Electronics.

[29] Kao KC, Hockham GA (1967). Dielectric-fibre surface waveguides for optical frequencies. Electromagnetic Wave Theory.

[30] Li G (2014). Transmission of Electromagnetic Waves in Softmaterial Waveguides. Applied Mechanics & Materials.

[31] Bezryadina A (2017). Nonlinear Self-Action of Light through Biological Suspensions. Physical Review Letters.

[32] Bear, M. F., Connors, B. W. & Paradiso, M. A. *Neuroscience: Exploring the brain* (3rd ed.). (Lippincott Williams & Wilkins, 2007).

[33] Schmitt FO, Bear RS, Palmer KJ (1941). X-ray diffraction studies on the structure of the nerve myelin sheath. Journal of Cellular Physiology.

[34] Zhang L, Zhu X, Ni Z (2012). Dielectric Parameter Solving Algorithm in Electrorotation Analysis of Spherical Cells. Journal of Southeast University (Natural Science Edition).

[35] Niu, Z. Q. The Self-electricfield Stress Exerted on Cell Membrane. Chinese *Journal of Biomedical Engineering*, 90–94 (1994).

[36] Grossman N (2017). Noninvasive Deep Brain Stimulation via Temporally Interfering Electric Fields. Cell.

[37] Thomas PK, Young JZ (1949). Internode lengths in the nerves of fishes. Journal of Anatomy.

[38] Malekabadi A, Charlebois SA, Deslandes D (2013). Parallel plate waveguide with anisotropic graphene plates: Effect of electric and magnetic biases. J. Appl. Phys..

[39] Ibrahim M, Butt AM, Berry M (1995). Relationship between myelin sheath diameter and internodal length in axons of the anterior medullary velum of the adult rat. Journal of the Neurological Sciences.

[40] Hildebrand C, Loeliger S, Bjartmar C, Karlsson M (1996). Sheath lengths of large motor axons in the ventral root L5 of neonatal and adult rats. Neuroscience Letters.

[41] Simpson AH (2013). Effect of limb lengthening on internodal length and conduction velocity of peripheral nerve. Journal of Neuroscience.

[42] Waxman SG (1980). Determinants of conduction velocity in myelinated nerve fibers. Muscle & Nerve.

[43] Knowles L (2017). The Evolution of Myelin: Theories and Application to Human Disease. Journal of Evolutionary Medicine.

[44] Rushton WA (1951). A theory of the effects of fibre size in medullated nerve. J Physiol.

[45] Friede RL (2017). The Significance of Internode Length for Saltatory Conduction: Looking Back at the Age of 90. J Neuropathol Exp Neurol.

[46] Salzer JL (1997). Clustering sodium channels at the node of Ranvier: close encounters of the axon-glia kind. Neuron.

[47] Meszler RM, Pappas GD, Bennett VL (1974). Morphology of the electromotor system in the spinal cord of the electric eel, Electrophorus electricus. J. Neurocytol..

[48] Saporta MA (2010). Reply: Internodal length variability of dermal myelinated fibres. Brain.

