# Supplementary material for: Experimental and Computational Studies on the Basic Transmission Properties of Electromagnetic Waves in Softmaterial Waveguides
Source: Sci Rep. 2018 Sep 14;8:13824. doi: 10.1038/s41598-018-32345-x (PMC6138705; doi:10.1038/s41598-018-32345-x)
Supplement: Supplementary file 1 — Supplementary Information [file 41598_2018_32345_MOESM1_ESM.pdf]

Supplementary Information

Experimental and Computational Studies on the Basic Transmission Properties of Electromagnetic Waves in Softmaterial Waveguides

Jingjing Xu[1, 2, †], Yuanyuan Xu[1, †], Weiqiang Sun[1, 2], Mingzhi Li[1, *], and Shengyong Xu[1, 2, *]

[1] Department of Electronics, School of Electronics Engineering and Computer Science, Peking University, Beijing 100871, P. R. China.

[2] Key Laboratory for Physics and Chemistry of Nanodevices, Peking University, Beijing 100871, P. R. China.

† These authors contributed equally to this work.
* Corresponding authors: mileslee@pku.edu.cn (MZL), xusy@pku.edu.cn (SYX)

Finite - difference time - domain method (FDTD) was utilized in computational simulation of this work. The FDTD technique is a well-developed computational method to simulate problems of electromagnetic (EM) fields with complex geometry and electromagnetic parameter distribution.

In this work, the spatial discrete step and time step were set to meet the following Courant Stability Condition:

$$v_p \Delta t \leq \frac{1}{\sqrt{\frac{1}{(\Delta x)^2} + \frac{1}{(\Delta y)^2} + \frac{1}{(\Delta z)^2}}}, \tag{1}$$

where $\Delta x = \Delta y = \Delta z$, $\Delta x = 2c_0 \Delta t$, and c_0 is the speed of EM waves in vacuum. In FDTD simulation, the most concerned parameter is the *electrical size*, which is defined as the ratio of geometric dimension of the model to the exciting signal wavelength. Our computing resource limited the *electrical size* in the range of 0.01 – 100 for this work. A uniaxial perfectly matched layer (UPML) was introduced as the absorbing boundary condition to restrict the computational domain.

We have performed calibrations to verify the FDTD code we developed in this work. The simulated results matched well with the results of EM field distributions in radiation of free space dipole, bistatic RCS (radar-cross section) of dielectric sphere as of theoretical formulas (e.g. Fresnel formula). **Fig. S1** gives the validation result from the test of the radiation of a free space dipole. It shows that the data calculated from our FDTD solutions and those from theoretical formula match with each other, proving the accuracy and applicability of the FDTD code.

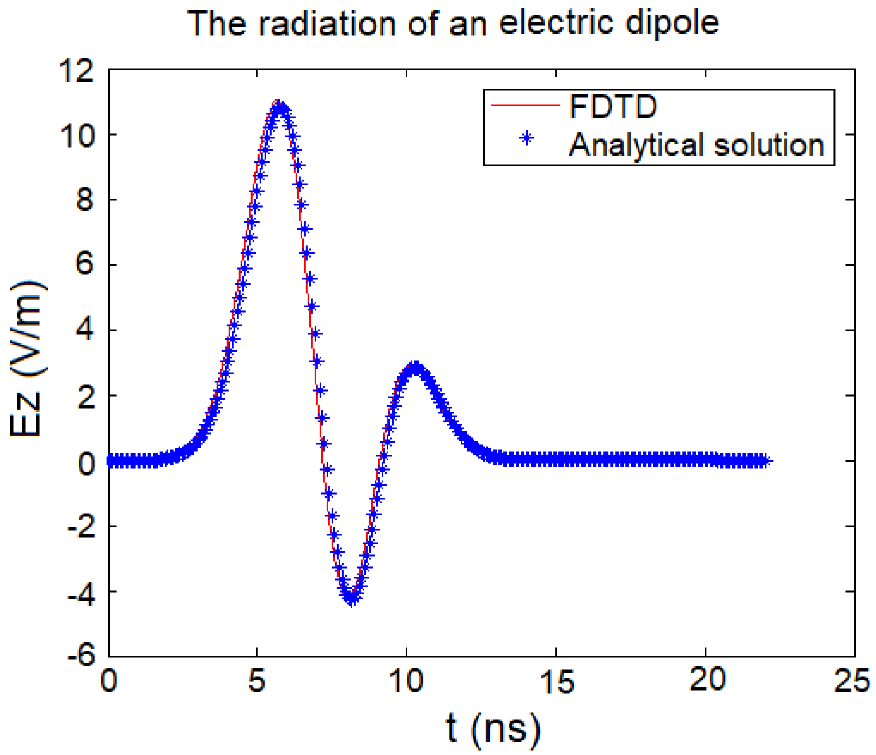

Figure S1. Test of the validation of our FDTD simulation results. The perfect match of solutions from our FDTD simulation and analytic method, indicating the accuracy and applicability of the FDTD method in this work.
